# E2F1 inhibition mediates cell death of metastatic melanoma

**DOI:** 10.1038/s41419-018-0566-1

**Published:** 2018-05-09

**Authors:** Florian Rouaud, Nedra Hamouda-Tekaya, Michaël Cerezo, Patricia Abbe, Joséphine Zangari, Veronique Hofman, Mickaël Ohanna, Baharia Mograbi, Najla El-Hachem, Zohra Benfodda, Alexandre Lebeau, Meri K. Tulic, Paul Hofman, Corine Bertolotto, Thierry Passeron, Jean-Sébastien Annicotte, Robert Ballotti, Stéphane Rocchi

**Affiliations:** 1 INSERM, U1065, team 12, Study of molecular mechanisms involved in pigmentation and melanoma using translational approaches, Centre Méditerranéen de Médecine Moléculaire (C3M), Nice, France; 2 Université Cote d’azur, UFR de Médecine, Nice, France; 3 Institute of Research on Cancer and Ageing of Nice (IRCAN), CNRS UMR7284, INSERM U1081, 06107 Nice, France; 4 Laboratoire de pathologie clinique et expérimentale et Hospital-related biobank (BB-0033-00025), Hôpital Pasteur, Nice, France; 5 Laboratoire de Détection, évaluation gestion des risques émergents et chroniques, Université de Nimes, Nîmes, France; 6 Centre Méditerranéen de Médecine Moléculaire (C3M), Team 1, INSERM U1065, Nice, France; 7 Service de Dermatologie, Hôpital Archet II, CHU de Nice, Nice, France; 8 CNRS, Institut Pasteur de Lille, UMR 8199–EGID, Université de Lille, 59000 Lille, France

## Abstract

Melanoma is one of the most lethal cancers when it reaches a metastatic stage. Despite advancements in targeted therapies (BRAF inhibitors) or immunotherapies (anti-CTLA-4 or anti-PD1), most patients with melanoma will need additional treatment. Thus, there is an urgent need to develop new therapeutical approaches to bypass resistance and achieve more prolonged responses. In this context, we were interested in E2F1, a transcription factor that plays a major role in the control of cell cycle under physiological and pathological conditions. Here we confirmed that E2F1 is highly expressed in melanoma cells. Inhibition of E2F1 activity further increased melanoma cell death and senescence, both in vitro and in vivo. Moreover, blocking E2F1 also induced death of melanoma cells resistant to BRAF inhibitors. In conclusion, our studies suggest that targeting the E2F1 signaling pathway may be therapeutically relevant for melanoma.

## Introduction

Cutaneous melanoma is one of the most lethal cancers among young adults. Melanoma has a high capability of rapid invasion and metastasizes to other organs. When lymph nodes metastase, the prognosis worsens considerably with a survival rate of 50% at 5 years. The increased knowledge about the molecular mechanisms of melanoma has revolutionized its treatment. Approximately half of melanomas express mutations in the protein kinase BRAF (such as BRAFV600E) that constitutively activate the mitogen-activated protein kinase (MAPK) pathway and result in a dysregulated proliferation irrespective of the presence of growth factors. The BRAF mutation constitutes a potential target for new anti-melanoma treatments, and the BRAF inhibitors vemurafenib and dabrafenib have demonstrated an improvement in both overall survival and progression-free survival^1^. Unfortunately, despite encouraging response rates seen using BRAF inhibitors, relapses usually occur within months after treatment^2^. Over the past 2 years, tremendous efforts have been directed toward understanding the molecular mechanisms of acquired BRAF inhibitor resistances^3,4^. Further, immunotherapies such as anti-CTLA-4 or anti-PD1 antibodies, which reactivate the immunity response of the patient, achieve durable responses or stable disease, but only in approximately 10 to 35% of patients^5^. Thus, there is an urgent need to develop new therapeutic approaches to bypass resistance and achieve more prolonged responses.

Cell proliferation is a tightly regulated process that comprises cyclins, cyclin-dependent kinases (CDKs), transcription factors, and CDK inhibitors^6^. The E2F1 transcription factor plays a major role in the control of cell cycle, in physiological and pathological conditions^7^. Deciphering the bona fide target genes of E2F1 demonstrated the key roles for this transcription factor in the regulation of cellular and tissue functions. Indeed, apoptosis, senescence, and glucose homeostasis are important mechanisms finely tuned by E2F1. Interestingly, recent data demonstrated that the overexpression of this factor is found in several types of cancers^8^. Altogether, these data suggest E2F1 as a potential therapeutic target for cancer cells. While E2F proteins, in particular E2F1, have emerged as critical players in melanoma development^9–11^, our mechanistic understanding of its regulation and function remains limited. Here, we report a key role for E2F1 in the control of melanoma cell death and drug sensitivity. E2F1 is highly expressed in melanoma cells. Depletion of E2F1 using small interfering RNA (siRNA) or pharmacological blockade of E2F activity further increased melanoma cell death and senescence, both in vitro and in vivo. Death and senescence induced by inhibition of E2F1 are as a result of p53 and p27 activation. Moreover, blocking E2F1 also induced death of melanoma cells resistant to BRAF inhibitors, and E2F1 inhibition increases sensitivity of melanoma cells to BRAF inhibitors. Our studies suggest that targeting the E2F1 signaling pathway may be therapeutically relevant for treatment of melanoma patients.

## Results

### E2F1 is overexpressed in melanoma

**Fig. 1:**
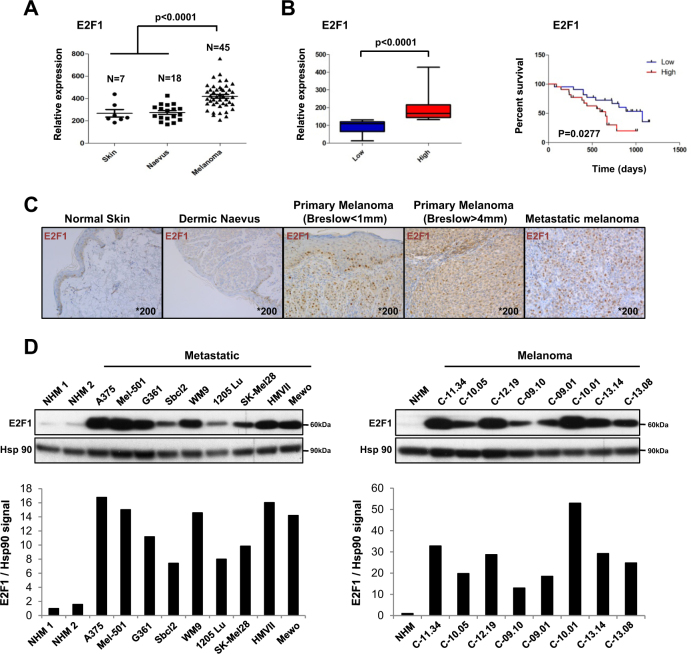
E2F1 is overexpressed in melanoma. **a** Level of E2F1 expression by microarray in healthy skin (*n* = 7), nevi (*n* = 18), and melanoma (*n* = 45) (GEOD3189^12^) (boxplots, Mann–Whitney test). **b** Survival of melanoma patients with high and low (above or below the median, respectively) *E2F1* mRNA. Gene expression data of 44 metastatic melanoma tissues^13^ were used to define high and low expressor groups (boxplots, Mann–Whitney test) and to generate Kaplan–Meier curves (log-rank test). **c** Representative immunostaining of E2F1 in normal skin and in different melanoma samples. **d** E2F1 expression in different melanoma cells and in normal human melanocytes (NHM) analyzed by western blot. HSP90 was used as A loading control. Signals were quantified using the Image J Software

Using publically available microarray data^12^, we analyzed E2F1 expression and detected increased *E2F1* mRNA levels in human melanoma biopsies compared to healthy skin and naevus (Fig. 1a). Interestingly, in a cohort of patients, followed in a clinic for 3 years after excision of metastatic lesions^13^, those with high E2F1 showed significantly lower survival (Fig. 1b). Using immunohistological analysis of human biopsies, we detected E2F1 staining in primary melanoma, with a robust expression in metastatic melanoma. E2F1 protein levels were not detected in non-cancerous tissues including skin and naevi (Fig. 1c and Table 1). By probing a panel of primary and metastatic melanoma cell lines and human melanocytes, we found that E2F1 is also strongly expressed in different melanoma cell lines and in melanoma cells freshly isolated from patients (Fig. 1d). Altogether, these findings confirm that E2F1 is highly expressed in melanoma cells.

**Table 1:** The intensity and cytoplasm localization of the E2F1 staining in normal skin and in different melanoma samples were analyzed and quantified by two pathologists (*n* = 3/condition)

Histology	% of E2F1-positive cells	Intensity of E2F1 labeling
Normal skin	Neg.	Neg.
Dermic naevus	Neg.	Neg.
Dermic naevus	Neg.	Neg.
Primary melanoma (Breslow <1 mm)	20%	Weak (nucl)
Primary melanoma (Breslow >4 mm)	30%	Weak (nucl)
Metastatic melanoma #1	40%	Moderate to strong (nucl and cyto)
Metastatic melanoma #2	50%	Moderate to strong (nucl and cyto)

### Inhibition of E2F1 has an anti-proliferative effect on melanoma cells

**Fig. 2:**
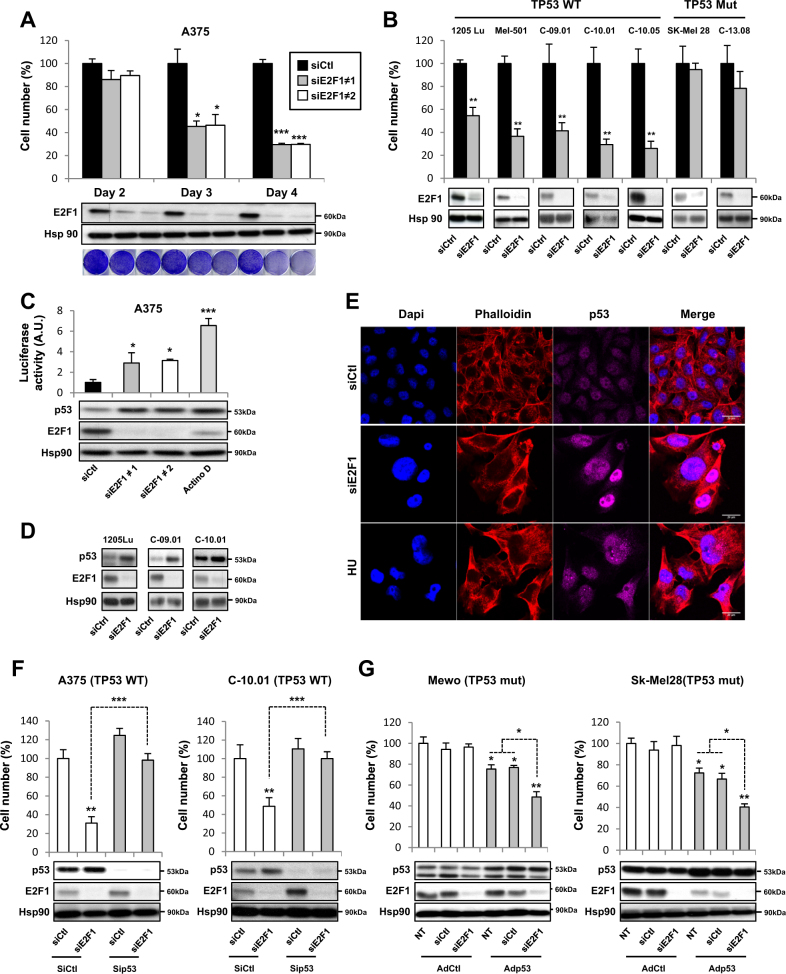
Inhibition of E2F1 by RNAi depletion decreases cell viability in melanoma cells in p53 dependent-manner. **a** The melanoma cell line A375 was transfected with control siRNA (siCtl 50 nM) or two different E2F1 siRNA (siE2F1 50 nM). At the indicated times, viable cells were counted using the trypan blue dye exclusion method. The results are expressed as percentages of control. In parallel, a crystal violet stain was used under the same conditions. **b** Indicated melanoma cell lines 1205Lu, Mel-501, or human melanoma cells freshly isolated from tumors were transfected with siCtl (50 nM) or siE2F1 (50 nM). After 4 days, viable cells were counted using the trypan blue dye exclusion method. The results are expressed as percentage of the control. **c** A375 cells were transfected with siCtl (50 nM) or siE2F1 (50 nM), and after 24 h, they were transfected with a p53-responsive promoter reporter (PG13-luc). After 24 h, luciferase activity was measured and normalized to β-galactosidase activities. p53 activator, actinomycin D (10 µg/ml), was used as a positive control. For **a**–**c**, cells were lysed and analyzed by western blotting using the indicated antibodies. HSP90 was used as a loading control. **d** 1205Lu melanoma cell line or human melanoma cells freshly isolated from tumors were transfected with siCtl or siE2F1 for 4 days. E2F1 and p53 levels were determined by western blot analysis. HSP90 was used as a loading control. **e** Immunofluorescence pictures of A375 melanoma cells transfected with siCtl or siE2F1. p53 was labeled with specific antibody (magenta), DNA was visualized with DAPI (blue), and phalloidin was visualized in red. Hydroxyurea (HU) was used as a positive control. A representative picture is shown. **f** A375 melanoma cells and human melanoma cells freshly isolated from tumors were co-transfected with siCtl or siE2F1 and siRNA against p53 (sip53 50 nM). After 4 days, viable cells were counted using the trypan blue dye exclusion method. In parallel, cells were lysed and analyzed by western blot using the indicated antibodies. HSP90 was used as a loading control. **g** Mewo and SK-Mel 28 melanoma cells were infected with adenovirus encoding a WT form of p53 (Adp53) or with a control adenovirus (AdCtl). After 24 h, cells were transfected with siE2F1. After 4 days, viable cells were counted using the trypan blue dye exclusion method. In parallel, cells were lysed and analyzed by western blotting using the indicated antibodies. HSP90 was used as a loading control. The results are expressed as percentages of the control and data are means ± SD of three independent experiments performed in triplicate. *P < 0.05, **P < 0.01, and ***P < 0.001

To assess whether E2F1 modulates the proliferation of melanoma cells, we developed two complementary approaches to inhibit E2F1 expression and/or activity. First, depletion of *E2F1* by two different siRNA resulted in the inhibition of cellular viability in a time-dependent manner (Fig. 2a). These results were replicated using several melanoma cells with different mutational status (Fig. 2b). We observed that the genetic inhibition of E2F1 induced loss of viability of these cancer cells. Interestingly, *TP53*-mutated cell lines (Sk-Mel 28 and the patient cells C-13.08) were resistant to E2F1 inhibition (Fig. 2b). In the complementary fashion, the use of pharmacological inhibitors of E2F1 and E2F4, HLM006474^9^, demonstrated the same effect on inhibition of cell viability (Supp Figure 1A and B). In parallel, we have evaluated some relevant E2F1 target genes by real-time quantitative reverse transcription PCR (qRT-PCR) after inhibition of E2F1 using siRNA or pharmacological inhibitor (Supp Figure 1C). Some genes such as *BIRC5*, *CDC6*, or *BRCA1* are inhibited in the same way by siE2F1 and HLM006474 and other genes such *BCL2* or *C-jun* are inhibited only by siE2F1.

We observed that inhibition of E2F1 does not affect E2F4 expression (Supp Figure 1D), suggesting a specificity of siRNA for E2F1. Interestingly, in the cohort of Bogunovic’s patients^13^, patients with high E2F4 did not have lower survival rates (Supp Figure 1E). We next observed that normal melanocytes and keratinocytes were resistant to the HLM006474 inhibitor (Supp Figure 1F). Finally, we tested HLM006474 in mice using xenograft experiments. Six days after injection of A375 in immunodeficient mice, we treated them with the vehicle or HLM006474 (2 mg per day per mouse) by intraperitoneal injection for 8 days and we followed their tumor development. Mice treated with HLM006474 demonstrated significantly reduced tumor growth compared to vehicle-treated animals (Supp Figure 1G). A strong decrease in the tumor weight and size were observed in mice treated with HLM006474 (Supp Figure 1H).

Since the genetic inhibition of E2F1 in melanoma cells that are mutated for p53 does not affect their viability, we hypothesized an implication of the p53 pathway. To test this, we performed different sets of experiments. First, we used a construct of p53-responsive elements fused to the luciferase reporter and observed that inhibition of E2F1 by siRNA led to transcriptional activation of p53 (Fig. 2c). This was confirmed by Western blot in two separate melanoma cell lines (A375 and 1205Lu) as well as in primary human melanoma cells (Fig. 2d). Interestingly, HLM006474 induced the same effect on p53 expression level (Supp Figure 2A), and tumors of HLM006474-treated mice had increased levels of p53 (Supp Figure 2B). Immunofluorescence experiments revealed that inhibition of E2F1 led to an increase expression and a nuclear localization of p53 (Fig. 2e and Supp Figure 2C).

To further decipher the functional contribution of p53 in E2F1-mediated control of melanoma cell proliferation, we silenced p53 expression in A375 cells and human melanoma cells freshly isolated from patient. Blocking p53 expression abrogated the decreased viability induced by E2F1 inhibition (Fig. 2f). Conversely, we performed rescue experiments in p53-mutated Mewo and SK-Mel 28 cells using an adenoviral approach. Although no effects on cell viability was observed when cells were transduced with the control adenovirus, wild-type p53 overexpression sensitized Mewo and SK-Mel 28 cells to E2F1 inhibition and reduced their cellular viability (Fig. 2g).

Collectively, these results suggest that the effects of E2F1 inhibition on melanoma cell death requires p53 activation and demonstrates that the E2F1 pharmacological inhibition may control tumor development in mice.

### E2F1 inhibition induces cell cycle arrest and apoptosis

**Fig. 3:**
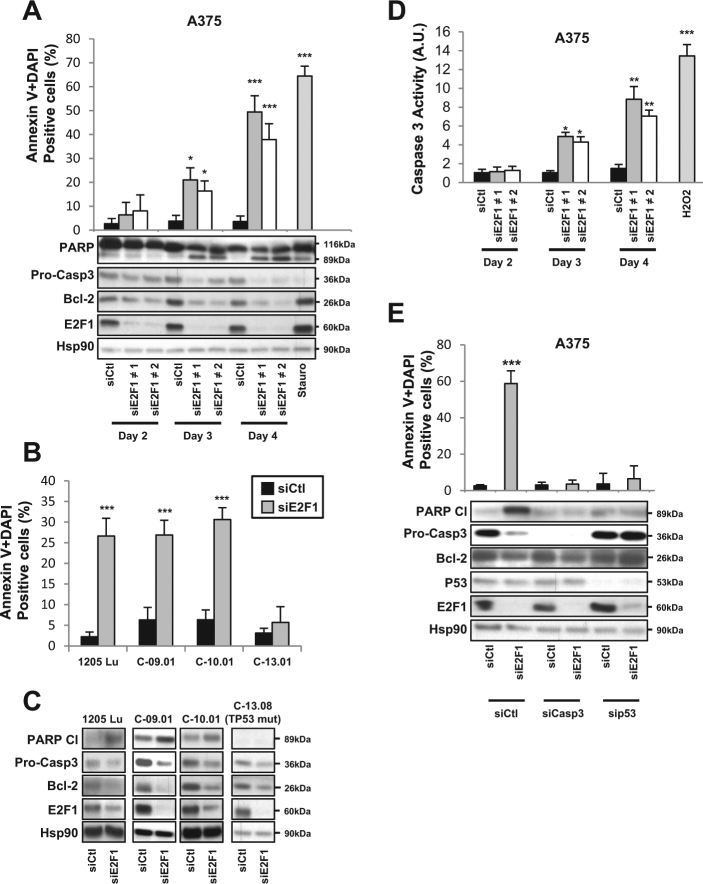
Inhibition of E2F1 induces p53-dependent apoptosis. **a** A375 melanoma cells were transfected with siCtl (50 nM) or two different siE2F1 (50 nM) for indicated times. At the end of experiment, cells were co-stained with Annexin V and DAPI to detect dead cells by flow cytometry. Staurosporine was used as a positive control. In parallel, cells were lysed and analyzed by western blotting using with indicated antibodies. HSP90 was used as a loading control. **b**, **c** 1205Lu melanoma cell lines or human melanoma cells freshly isolated from tumors were transfected with siCtl or siE2F1. **b** After 4 days, cells were co-stained with Annexin V and DAPI to detect dead cells by flow cytometry. **c** In parallel, cells were lysed and analyzed by western blotting using indicated antibodies. HSP90 was used as a loading control. **d** A375 melanoma cells transfected with siCtl or siE2F1 for indicated times. **d** Caspase-3 activity was determined by fluorometric assay. H_2_O_2_ was used as positive control. **e** A375 melanoma cells were transfected with siRNA against caspase-3 (siCasp3 50 nM) or p53 (sip53 50 nM) or co-transfected with siCtl or siE2F1 for 4 days. Cells were co-stained with Annexin V and DAPI to detect dead cells by flow cytometry. In parallel, cells were lysed and analyzed by western blotting using the indicated antibodies. HSP90 was used as a loading control. For **a**, **d**, **e**, the results are expressed as percentages. Data are means ± SD of three independent experiments. *P < 0.05, **P < 0.01, and ***P < 0.001

To further understand the molecular mechanisms linking E2F1 and melanoma cell viability, we measured cell cycle phases by propidium iodide (PI) labeling in A375 melanoma cells. Inhibiting E2F1 by siRNA induced an arrest in the G1 phase and HLM006474 induced an arrest in the G2/M phase (Supp Figure 3a). We confirmed these results by immunoblot (data not shown). We also determined apoptosis by performing a double Annexin V + DAPI (4',6-diamidino-2-phenylindole) labeling in A375 melanoma cells. Inhibiting E2F1 by siRNA or HLM006474 strongly increased the number of Annexin V + DAPI-positive cells, characteristic of apoptosis (Fig. 3a and Supp Figure 3B). This effect was associated with decreased levels of the anti-apoptotic protein Bcl-2, increased poly (ADP-ribose) polymerase (PARP) cleavage, and decreased zymogen form of caspase-3 (Fig. 3a and Supp Figure 3B). These results were confirmed in other melanoma cells (Fig. 3b, c and Supp Figure 3C and D) and tumors of mice injected with the E2F inhibitor (Supp Figure 3E). As expected, caspase-3 activation was observed after E2F1 inhibition (Fig. 3d). Flow cytometry measurements of apoptosis after silencing caspase-3 further demonstrated its contribution to apoptosis following E2F1 inhibition (Fig. 3e). Blocking caspase-3 or p53 by siRNA inhibited the cleavage of PARP (Fig. 3e), confirming that both caspase-3 and p53 are necessary to induce apoptosis upon E2F1 inhibition.

### E2F1 inhibition leads to a p53-dependent program of senescence

**Fig. 4:**
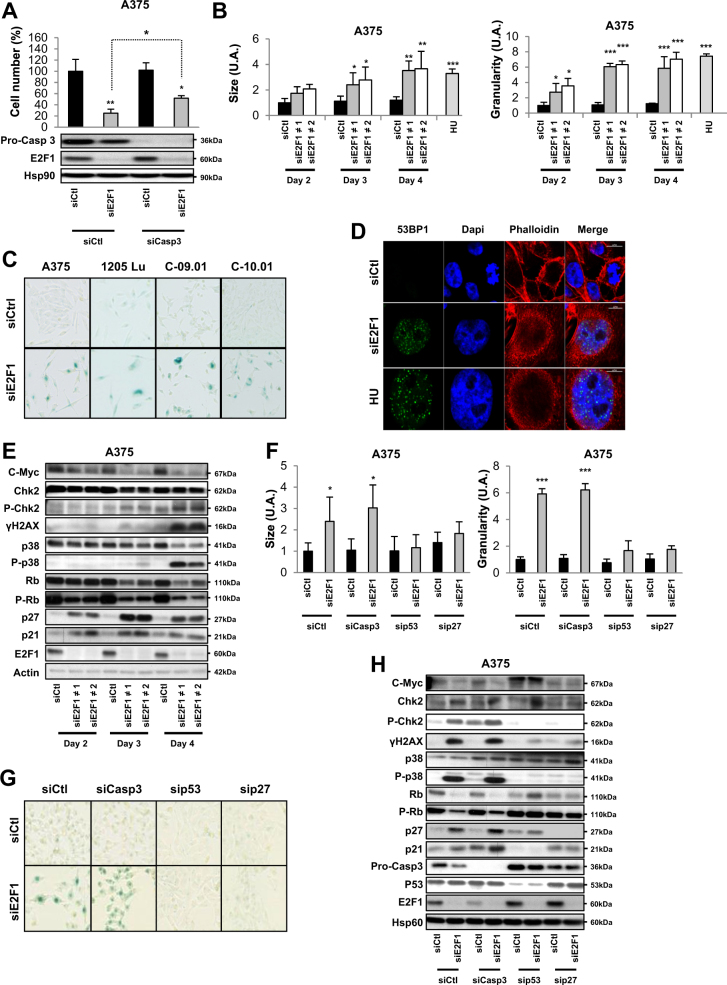
Inhibition of E2F1 induces senescence with activation of DNA damage in a p53-dependent and p27-dependent manner. **a** A375 melanoma cells were co-transfected with siCasp3 and siCtl or siE2F1. After 4 days, viable cells were counted using the trypan blue dye exclusion method. The results are expressed as the percentage of control. In parallel, cells were lysed and analyzed by western blotting using with indicated antibodies. HSP90 was used as a loading control. **b** A375 melanoma cells were transfected with siCtl or two different siE2F1 for indicated times. Cells were then analyzed by flow cytometry for the relative size and cell granularity. Hydroxyurea (HU) was used as positive control. **c** Indicated melanoma cell lines or human melanoma cells freshly isolated from tumors were transfected with siCtl or siE2F1. After 4 days, cells were stained for SA-β-Gal activity. A representative picture is shown. **d** Immunofluorescence pictures of A375 melanoma cells transfected with siCtl or siE2F1 for 4 days. 53BP1 was labeled with specific antibody (green), DNA was visualized with DAPI (blue), and phalloidin was visualized with red. Hydroxyurea (HU) was used as a positive control. A representative picture is shown. **e** A375 melanoma cells were transfected with siCtl or two different siE2F1 for indicated times. Cell lysates were analyzed by western blotting using the indicated antibody. Actin was used as a loading control. One representative experiment of three is shown. **f**–**h** A375 melanoma cells were co-transfected with sip53 or siCasp3 or sip27 and siCtl or siE2F1. **f** Cells were analyzed by flow cytometry for the relative size and cell granularity. The results are expressed as percentage of control. **g** Cells were stained for SA-β-Gal activity. A representative picture is shown. **h** Cells were lysed and analyzed by western blotting using the indicated antibodies. HSP60 was used as a loading control. For **a**, **b**, **f**, the results are expressed as percentages of the control. Data are means ± SD of three independent experiments. *P < 0.05, **P < 0.01, and ***P < 0.001

Although we demonstrated that apoptosis contributed to the observed effect on cell proliferation, this process only partially restored the loss of cellular proliferation induced by the inhibition of E2F1 (Fig. 4a). This indicated that other mechanisms could contribute to reduced cell viability. Flow cytometry experiments demonstrated that the inhibition of E2F1 led to an increase in size and the granularity of melanoma cells (Fig. 4b and Supp Figure 4A and B), which are morphological characteristics of cellular senescence^14^. Inhibition of E2F1 increased the β-galactosidase activity of melanoma cells (Fig. 4c and Supp Figure 4C). The senescence program is associated with induction of DNA damage and increased 53BP1 foci in damaged cells. After inhibition of E2F1, immunofluorescence analysis confirmed increased 53BP1 foci in cells with altered E2F1 activity (Fig. 4d and Supp Figure 4D). The induction of DNA damage following the inhibition of E2F1 correlated with increased amount of phospho (p)-CHK2 and γH2AX protein, two markers of this process (Fig. 4e and Supp Figure 4E). We also observed p38 activation and a concomitant decrease in c-Myc and phosphorylated (p)-Rb, as well as an increase in p27 and p21 activation (Fig. 4e and Supp Figure 4E). These results were confirmed in other melanoma cells and in tumors of mice treated with E2F inhibitor (Supp Figure 4F and G).

We next determined whether p53 and p27 were key mediators of the senescence program triggered by E2F1 inhibition, and whether apoptosis and senescence were two independent mechanisms that contributed to reduce cell viability. siRNA-mediated inhibition of caspase-3 demonstrated increased size and granularity (Fig. 4f) and β-galactosidase staining (Fig. 4g) of melanoma cells. Moreover, these effects were associated with increased p21, p27, p-Chk2, and γH2AX protein but decreased p-Rb (Fig. 4h). In contrast, siRNA-mediated inhibition of p53 or p27 did not increase the size or the granularity of the cells (Fig. 4f), nor β-galactosidase activity (Fig. 4g) or p21, p27, p-Chk2, γH2AX, and p-Rb (Fig. 4h).

Our results indicate that the process of senescence induced by the inhibition of E2F1 is dependent on the p53 and p27 and that apoptosis and senescence may occur independently. Finally, we determined that siE2F4 are also able to induce death of melanoma cells by apoptosis. However, contrary to siE2F1, siE2F4 did not induce senescence (Supp Figure 5).

### E2F1 inhibition increases sensitivity of the melanoma cells to BRAF inhibitors and induces the death of the vemurafenib-resistant melanoma cells

**Fig. 5:**
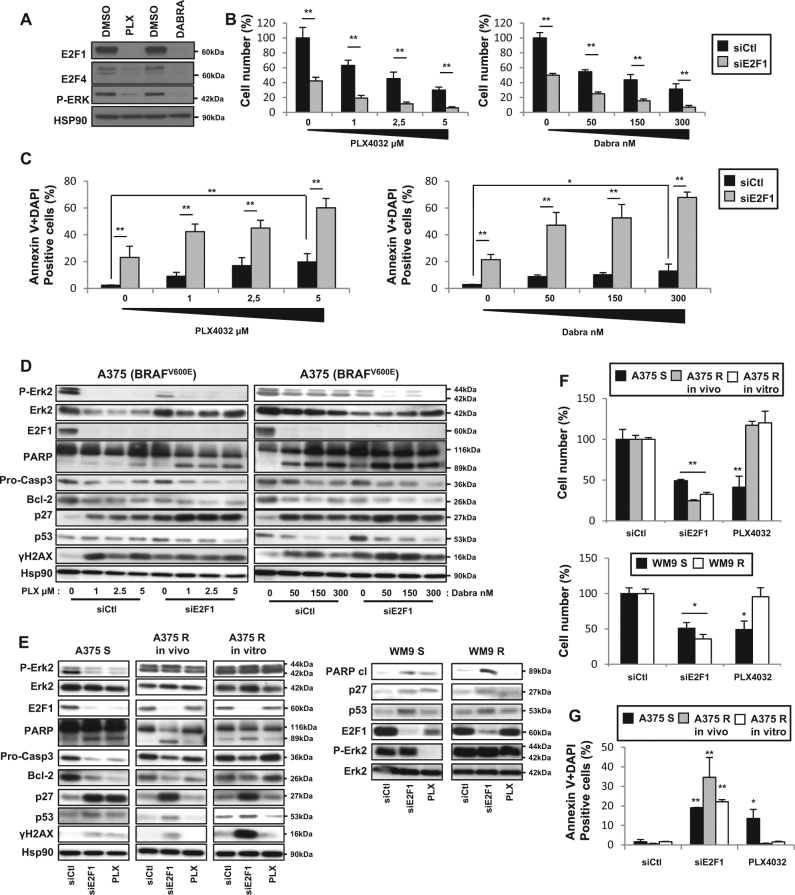
E2F1 inhibition sensitizes melanoma cells to targeted therapies and induces the death of BRAF inhibitor-resistant melanoma cells. **a** A375 melanoma cells were treated with DMSO or PLX4032 (5 μM) or dabrafenib (300 nM) for 2 days. Cells were lysed and analyzed by western blotting using the indicated antibodies. **b**–**d** A375 melanoma cells were transfected with siCtl or siE2F1, and 36 h later, cells were treated with BRAF inhibitors, PLX4032 (1, 2.5, or 5 µM) or dabrafenib (50, 150, or 300 nM) for 48 h. **b** Viable cells were counted using the trypan blue dye exclusion method. The results are expressed as the percentage of control. **c** Cells were co-stained with Annexin V and DAPI to detect dead cells by flow cytometry. **d** In parallel, cells were lysed and analyzed by western blotting using the indicated antibodies. HSP90 was used as a loading control. **e**–**g**. A375 melanoma cells sensitive (S) or resistant (R) to PLX4032 or WM9 sensitive (S) or resistant (R) to PLX4032 were transfected with siCtl or siE2F1 for 3 days or treated with PLX4032 for 48 h. **e** Viable cells were counted using the trypan blue dye exclusion method. The results are expressed as the percentage of control. **f** In parallel, cells were lysed and analyzed by western blotting using the indicated antibodies. HSP90 or Erk2 was used as a loading control. **g** A375 cells S and R were co-stained with Annexin V and DAPI to detect dead cells by flow cytometry. For **b**, **c**, **f**, **g**, data are means ± SD of three independent experiments. *P < 0.05 and **P < 0.01

We next wondered whether inhibiting E2F1 could potentiate the effects of the BRAF inhibitors, vemurafenib (PLX4032) and dabrafenib. We first assessed the effect of PLX4032 on E2F1 and E2F4 levels and we observed that BRAF inhibitors, PLX4032 and dabrafenib, reduce E2F1 and E2F4 levels (Fig. 5a). Second, we investigated whether E2F1 depletion acts synergistically with BRAF inhibitors to enhance its pro-apoptotic effect on melanoma cells. Whereas increasing doses of PLX4032 or dabrafenib or E2F1 depletion alone reduced cell viability (Fig. 5b), combination of BRAF and E2F1 inhibition resulted in increased cell death in BRAF mutant lines (Fig. 5b). Using double-labeling Annexin V + DAPI and flow cytometry, we observed a strong increase in the percentage of double-positive cells that are characteristic of apoptotic cell death, when combined treatments were used (Fig. 5c). To confirm this apoptosis, western blot analysis indicates that the combination of BRAF inhibitor treatment and E2F1 depletion drastically decreased the pro-form of the caspase-3 and Bcl-2 and led to increased PARP cleavage (Fig. 5d). We next used a series of different BRAF-resistant melanoma cells^15^. Intriguingly, the inhibition of E2F1 induced loss of cell viability of sensitive and BRAF inhibitor-resistant melanoma cells (Fig. 5e). In order to determine whether apoptosis could be responsible for the apparent cell death, western blot analysis indicated that E2F1 depletion decreases pro-form of the caspase-3 and Bcl-2, and an increase in PARP cleavage, p53 and p27 (Fig. 5f). To confirm this apoptosis, we quantified Annexin V + DAPI co-stained cells by flow cytometry and have shown that inhibition of E2F1 led to an increase in Annexin V + DAPI labeling in double-labeled cells that were resistant to PLX4032 (Fig. 5g). In parallel, we observed that E2F1 inhibition is also able to induce DNA damage in A375-resistant melanoma cells as indicated by increased expression of γH2AX (Fig. 5f). We next carried out experiments to evaluate the effects of E2F1 blocking in combination with BRAF inhibitor, PLX4032, in BRAF inhibitor-resistant melanoma cells. As presented in Supp Figure 6, we observed that co-inhibition of BRAF by PLX4032 and E2F1 by siRNA did not increase apoptosis (PARP cleavage and Annexin V-positive cells) or DNA damage (γH2AX) in comparison to E2F1 inhibition alone. These results indicate that BRAF inhibitors do not increase the death induced by inhibition of E2F1.

## Discussion

Metastatic melanoma is notoriously refractory to conventional cancer therapies and remains largely resistant to current targeted therapies. Our study has demonstrated that inhibition of E2F1 (transcription factor from E2F1 family) is effective in decreasing melanoma cell viability in both sensitive and resistant metastatic melanoma cells.

E2F1 is the most studied member of the family and presents several yet opposing roles in the control of cell growth and apoptosis, depending on the cell type or tissue contexts. E2F gene amplification has been described in various cancer cell lines and tumor types^11^. Further, higher levels of E2F1 are frequently associated with high-grade tumors and poor patient survival prognosis^13,16^. Our results are in agreement with those in the literature showing higher expression of E2F1 in metastatic melanoma and bring novel data regarding its mechanisms of effect, and, importantly, give an important insight into its potential beneficial role in resistive patients.

The role of E2F1 in apoptosis is contradictory. It has been reported that the ambivalent pro-apoptotic or anti-apoptotic function of E2F1 depends on the tissue type and/or cellular contexts. In our system, we confirm that inhibition of E2F1 using siRNA in melanoma cells leads to apoptotic cell death. This was demonstrated using multiple techniques including the number of Annexin V/DAPI-double-positive cells, PARP cleavage, activation of caspase-3, and anti-apoptotic protein Bcl-2. We also demonstrated that E2F1 inhibition in melanoma cells induces a cellular phenotype displaying several hallmarks of senescence, such as larger but flatter cells with more granulation and increased senescence-associated-β-gal (SA-β-Gal) activity. Analyzing the expression of proteins involved in the control of the cell cycle and senescence revealed increased p21^CIP1^ and p27^KIP1^ when E2F1 activity is lowered^17–20^. Our study also highlighted that the crucial role of p21 and/or p27 is the control of E2F1-dependent senescence.

The senescence program is often associated with DNA damage^14,21^. These observations prompted us to investigate the involvement and the role of DNA damage following E2F1 inhibition. In agreement with our hypothesis, we have shown that the senescence mediated by E2F1 inhibition engages a broad DNA damage response signaling pathway. In addition, we observed that inhibition of E2F1 in melanoma cells leads to the presence of 53BP1 foci and increased γH2AX, two mediators recruited and phosphorylated at DNA-damaged sites. We also observed the activation of the critical mediator of cellular responses to DNA damage, CHK2. Noteworthy, although we cannot rule out the existence of DNA single-strand breaks, engagement of the γH2AX/53BP1/CHK2 cascade in E2F1-inhibited cells strongly indicates that the type of DNA damage is mainly double-strand breaks. Recent data described that reactive oxygen species (ROS) play a crucial role in maintaining DNA damage, which is necessary for a stable arrest of the proliferation that is characteristic of a senescence program^22^. As shown here, E2F1 inhibition leads to increased ROS production, which could be the initiating factor of this program. However, our data using *N*-acetyl cysteine to prevent ROS induction did not inhibit cell viability and induction of senescence mediated by siE2F1, suggesting that ROS are not involved in these process (Supp Figure 3J and data not shown). Interestingly, these senescence program and DNA damage response are also independent of apoptosis mechanism.

One protein that can mediate these different effects is the tumor suppressor p53. Indeed, p53 functions as a transcriptional regulator of genes involved in cell cycle arrest, autophagy, senescence, and apoptosis pathways. First, we demonstrated that the inhibition of E2F1 increases p53 expression and activity. Second, melanoma cells harboring a mutated *TP53* gene exhibited no modification of melanoma viability after E2F1 inhibition. Identical results were obtained when we invalidated endogenous p53 in melanoma cells. This effect is not associated with apoptosis, DNA damage, or senescence induction. Interestingly, rescuing the expression of TP53 in p53-knockdown cells restores E2F1 inhibition effects, further indicating the key function of p53 in mediating the effects of E2F1 inhibition in our cellular models. In addition, our studies suggest that E2F1 functions upstream of p53 in the crosstalk between these two transcription factors in the context of apoptosis and senescence.

In parallel, we have evaluated the anti-melanoma activity of the E2F inhibitor HLM006474 in intact melanoma cells and in a mouse model of melanoma xenograft. As previously described, HLM006474 is not specific for E2F1 as it can also inhibit E2F4^9^. In melanoma cells, HLM006474 induces similar effect as observed with specific E2F1 siRNA. As blocking E2F4 also inhibited melanoma cell viability, it is not excluded that the effects of HLM006474 could also be mediated by E2F4 inhibition. Further studies will be necessary to determine the respective contribution of E2F1 and E2F4 in the effects of HLM006474 in melanoma cells.

Importantly, we found that short-term administration of HLM006474 dramatically reduces the development of melanoma tumors in mice. In addition, HLM006474 induces no apparent toxicity, as the body weight and overall appearance of mice given an HLM006474 regimen were not different from controls. To determine whether the molecular events observed in melanoma cells were also found in xenograft mice, we performed western blot analysis which indicated p53 activation induced by HLM006474, induction of apoptosis, and DNA damage in tumors of treated mice. In mice, we saw no effect on normal cells (melanocytes/keratinocytes) demonstrating specificity of E2F inhibitor in melanoma cells.

The development of resistance to BRAF inhibitors is a major obstacle in targeted therapy for melanoma. Our data clearly indicate that E2F1 inhibition potentiates the anti-proliferative and pro-apoptotic effects of BRAF inhibitors, PLX4032 and dabrafenib, in melanoma cell lines sensitive to BRAF inhibitor. The restoration of p53 activity in melanoma p53-deficient cells is effective in increasing the sensitivity of melanoma cells to the BRAF-MAPK inhibitor pathway^23,24^. Given that the effects of E2F1 inhibition on cell viability are p53 dependent, we postulate that these effects on BRAF inhibitor sensitivity are mediated through p53 activation. This hypothesis is currently tested in our laboratory. Furthermore, our data also indicate that BRAF inhibitors reduce E2F1 expression and this is likely to be due to G1/S arrest observed in response to these compounds. We next performed experiments in BRAF inhibitor-resistant melanoma cells. There have been multiple mechanisms previously proposed^25^. We failed to detect classical secondary mutations in NRAS, KRAS, or MEK, or elevated levels of BRAF or alternatively splice isoforms^15^. E2F1 inhibition decreased the viability of melanoma cells with acquired resistance to BRAF inhibitor through the induction of apoptosis and DNA damage. We were not able to determine if the loss of E2F1 led to senescence process in the resistant cells due to the large cell death observed. Further, our experiments indicate that BRAF inhibitors do not increase the death induced by inhibition of E2F1 in melanoma cells resistant to BRAF inhibitors.

Taken together, E2F1 loss enhances sensitivity of melanoma cells to targeted therapies and induces the death of BRAF inhibitor-resistant melanoma cells. Collectively, these data suggest that E2F1 is a critical mediator of melanoma drug sensitivity and may serve as an important target for novel therapeutic strategies. Further, this study provides compelling data to support future evaluation of HLM006474 in clinical trials and strengthen the hypothesis that E2F inhibitors could be useful as a new therapeutic approach for melanoma treatment.

## Materials and methods

### Reagents and antibodies

Trypan blue, Dulbecco's modified Eagle's medium, RPMI, trypsin, and siRNA were purchased from Life Technologies. Annexin V and DAPI were purchased from Roche Applied Science. Dabrafenib and PLX4032 were purchased from Euromedex. Antibodies against HSP90, E2F1, E2F4, Rb, and phospho-Rb were from Santa Cruz Biotechnology (TEBU, Le Perray en Yvelines, France). Antibodies against PARP, pro-caspase-3, p38, phospho-p38, MDM2, Chk2, p-Chk2, p21, and p27 were from BD Bioscience (Pont de Claix, France) and anti-H2AX was from Merck Millipore (Fontenay-sous-Bois, France).

The E2F1 siRNA number 1 corresponds to a Smart pool (Dharmacon ref: L-003259-00-0005) and the siRNA number 2 was purchassed to Ambion (Ref: 4390824).

### Cell cultures

Melanocytes and fibroblasts were prepared as described^26^. Different melanoma cell lines were purchased from American Tissue Culture Collection. Resistant melanoma cell lines A375 and WM9 were a gift from Pr. Marchetti and as described^15^ (see Supplemental Materials and methods). Fresh sterile tissues were obtained as surgical waste from patients diagnosed for metastatic melanoma at the Nice CHU hospital and treated as reported^27^.

### siRNA transfection

Transfection of duplex siRNAs was as described^28^.

### Trypan blue exclusion assay

Trypan blue staining was conducted as described^29^.

### Luciferase assay

Luciferase assays were conducted as described^29^.

### Western blot assay

Western blot analyses were performed as described^30^.

### Immunofluorescence

Immunofluorescence staining was performed as described^31^.

### SA-β-Gal assay

SA-beta-Gal Kit (Cell Signaling Technology) was used to histochemically detect β-galactosidase activity.

### Flow cytometry analysis

Cells were stained with DAPI and Annexin V or Cell ROX Deep Red and analyzed using fluorescence-activated cell sorter (MACSQuant Analyzer) and MACSQuantify software as described^32^.

### In vivo murine cancer model

Animal experiments were carried out in accordance with French law and were approved by a local institutional ethical committee CIEPAL. A375 cells were subcutaneously inoculated in the left dorsal side of 6-week-old female athymic nude nu/nu mice (Harlan, Gannat, France). The tumor size was assessed using calipers, and the volume was calculated according to the formula: tumor volume (mm^3^) = tumor width × length^2^ × 0.5. At the end of the experiment, mice were killed, tumors were excised, and then analyzed.

### Statistical analysis

Unless stated otherwise, experiments shown were representative of at least three independent experiments. All data were presented as means ± standard deviation. Statistical analysis was performed using the Student's *t* test: *P < 0.05 vs. control, **P < 0.01 vs. control, and *** *P* < 0.001 vs. control deemed statistically significant. For microarray analysis, Mann–Whitney test was performed.

## Electronic supplementary material

Supp figure 1

Supp figure 2

Supp figure 3

Supp figure 4

Supp figure 5

Supp figure 6

Supplemental materials
