# Supplementary material for: E2F1 inhibition mediates cell death of metastatic melanoma
Source: Cell Death Dis. 2018 May 9;9(5):527. doi: 10.1038/s41419-018-0566-1 (PMC5943238; doi:10.1038/s41419-018-0566-1)
Supplement: Supplementary file 1 — Supp figure 1 [file 41419_2018_566_MOESM1_ESM.pptx]

## Slide 1
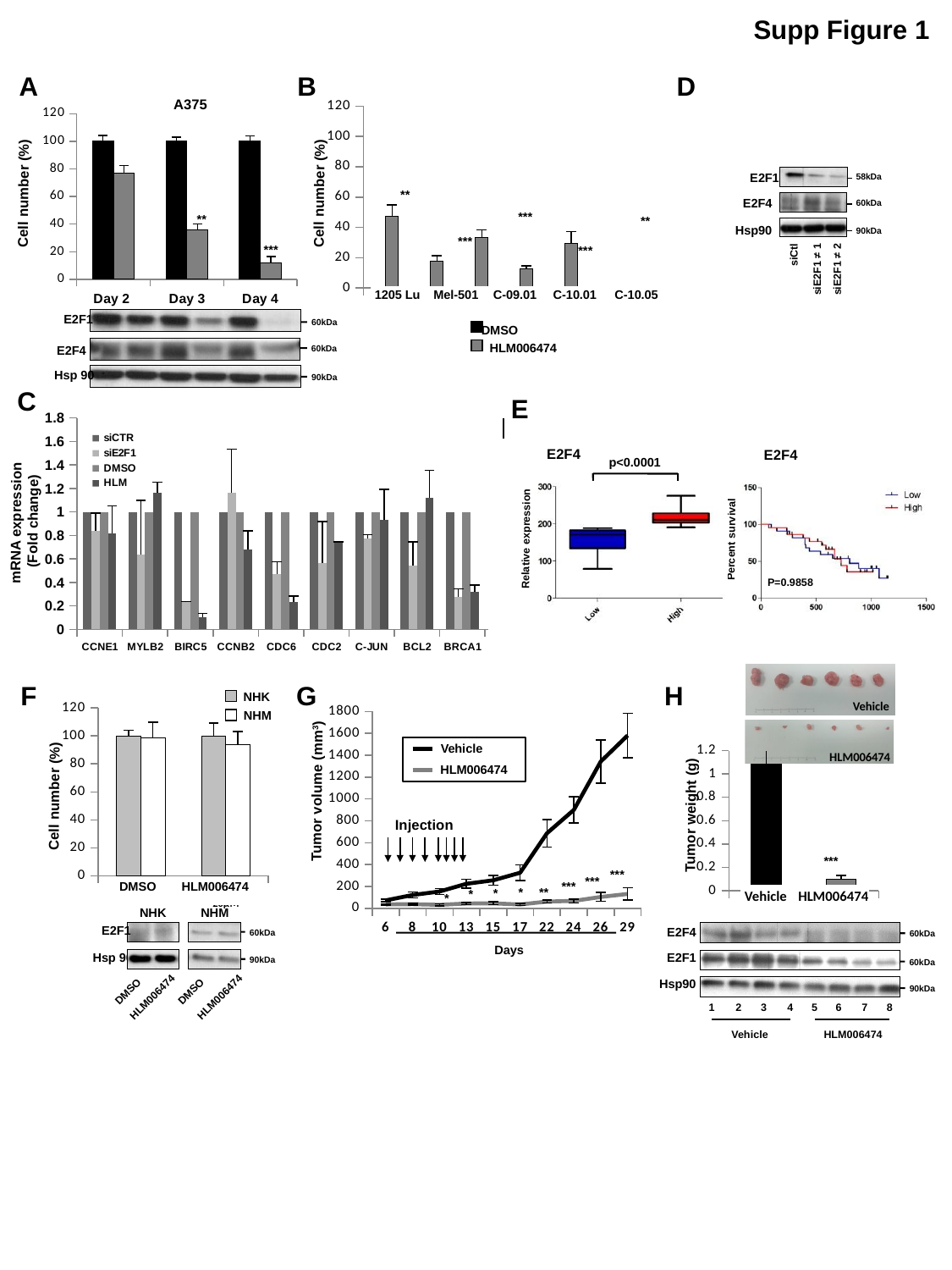

Supp Figure 1
A
B
D
A375
### Chart
| Category | | |
|---|---|---|
### Chart
| Category | | |
|---|---|---|
| Day 2 | 100.0 | 76.99530516431926 |
| Day 3 | 100.0 | 35.88516746411479 |
| Day 4 | 100.0 | 12.206572769953048 |E2F1
E2F4
Hsp90
siCtl
siE2F1 ≠ 1
siE2F1 ≠ 2
58kDa
Cell number (%)
Cell number (%)
**
60kDa
***
**
**
90kDa
***
***
***
1205 Lu
Mel-501
C-09.01
C-10.01
C-10.05
E2F1
60kDa
DMSO
HLM006474
E2F4
60kDa
Hsp 90
90kDa
C
E
### Chart
| Category | siCTR | siE2F1 | DMSO | HLM |
|---|---|---|---|---|
| CCNE1 | 1.0 | 0.8412966876528211 | 1.0 | 0.81883762368236 |
| MYLB2 | 1.0 | 0.6399712553555442 | 1.0 | 1.164997330281412 |
| BIRC5 | 1.0 | 0.229802149312256 | 1.0 | 0.10166331286301501 |
| CCNB2 | 1.0 | 1.1605527270642073 | 1.0 | 0.6796705867115962 |
| CDC6 | 1.0 | 0.469003824522751 | 1.0 | 0.23208043067608103 |
| CDC2 | 1.0 | 0.5638248064184642 | 1.0 | 0.7353097536128421 |
| C-JUN | 1.0 | 0.7717272371493092 | 1.0 | 0.93150278718881 |
| BCL2 | 1.0 | 0.5441914284653201 | 1.0 | 1.121467760820458 |
| BRCA1 | 1.0 | 0.2772227130549551 | 1.0 | 0.3223854633308552 |
| TP53 | 1.0 | 1.5774708821493977 | 1.0 | 1.4985351171667731 |E2F4
E2F4
p<0.0001
mRNA expression
(Fold change)
Relative expression
Percent survival
P=0.9858
F
G
H
NHK
Vehicle
### Chart
| Category | | |
|---|---|---|
| DMSO | 100.0 | 98.65771812080503 |
| Inhibitor E2F 20µM | 100.0 | 93.87755102040815 |
### Chart
| Category | | |
|---|---|---|
| 6 | 72.45034838142847 | 35.27438346166653 |
| 8 | 121.49097114166659 | 37.88538059666658 |
| 10 | 153.57684589466658 | 31.267426373999953 |
| 13 | 224.057533379 | 43.6107963551852 |
| 15 | 256.0597923044447 | 45.21068162499999 |
| 17 | 324.7675437693333 | 34.66977185633333 |
| 22 | 686.7717495256682 | 61.713065122916646 |
| 24 | 900.261769172 | 67.37600384933333 |
| 26 | 1342.590607367 | 103.66522442566671 |
| 29 | 1581.1520915099998 | 131.0925518466667 |NHM
Vehicle
### Chart
| Category | |
|---|---|
| Labra | 1.081 |
| Inhi E2F | 0.09900000000000002 |HLM006474
HLM006474
Tumor volume (mm3)
Cell number (%)
Tumor weight (g)
Injection
***
***
***
***
DMSO
HLM006474
**
*
*
*
Vehicle
HLM006474
*
NHK
NHM
E2F1
E2F4
60kDa
60kDa
Days
E2F1
Hsp 90
90kDa
60kDa
Hsp90
90kDa
DMSO
DMSO
HLM006474
HLM006474
1
2
3
4
5
6
7
8
HLM006474
Vehicle
