# Supplementary material for: E2F1 inhibition mediates cell death of metastatic melanoma
Source: Cell Death Dis. 2018 May 9;9(5):527. doi: 10.1038/s41419-018-0566-1 (PMC5943238; doi:10.1038/s41419-018-0566-1)
Supplement: Supplementary file 2 — Supp figure 2 [file 41419_2018_566_MOESM2_ESM.pptx]

## Slide 1
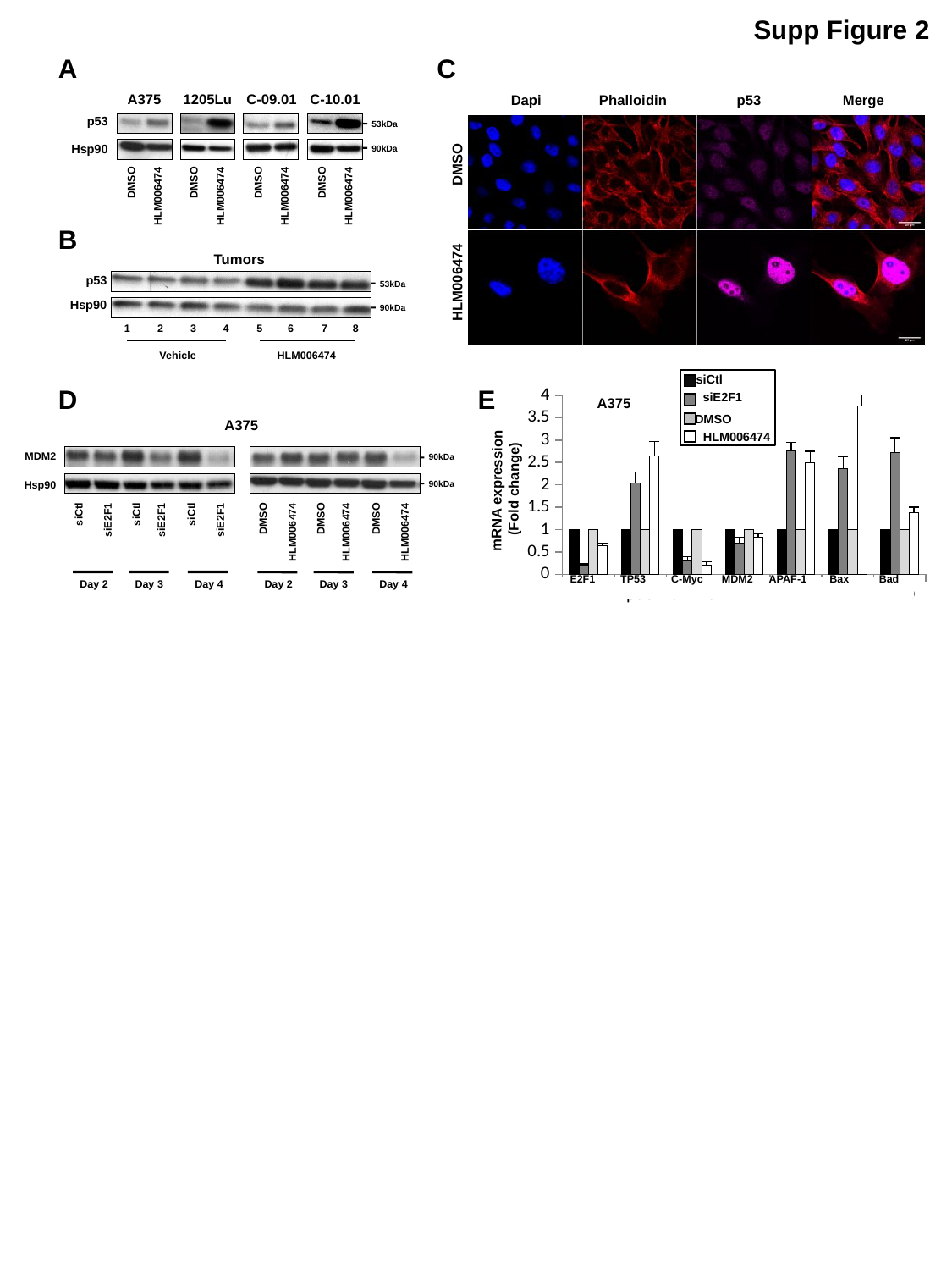

Supp Figure 2
A
C
A375
1205Lu
C-09.01
C-10.01
Dapi
Phalloidin
p53
Merge
p53
53kDa
Hsp90
90kDa
DMSO
DMSO
DMSO
DMSO
DMSO
HLM006474
HLM006474
HLM006474
HLM006474
B
Tumors
p53
HLM006474
53kDa
Hsp90
90kDa
1
2
3
4
5
6
7
8
HLM006474
Vehicle
siCtl
D
E
### Chart
| Category | Moyenne A375 siCtrl | Moyenne A375 siE2F1 | Moyenne A375 DMSO | Moyenne A375 Inh |
|---|---|---|---|---|
| E2F1 | 1.0 | 0.21354889248026707 | 1.0 | 0.6394479725133682 |
| p53 | 1.0 | 2.0364283156258582 | 1.0 | 2.647691516879414 |
| C-MYC | 1.0 | 0.30137315163991707 | 1.0 | 0.20513904194721702 |
| MDM2 | 1.0 | 0.6967112466019401 | 1.0 | 0.8305164840020799 |
| APAF1 | 1.0 | 2.76704410936772 | 1.0 | 2.496733187639914 |
| BAX | 1.0 | 2.3624190457229783 | 1.0 | 3.7609933070751653 |
| BAD | 1.0 | 2.7285625147521912 | 1.0 | 1.382942345888402 |siE2F1
A375
DMSO
A375
HLM006474
MDM2
90kDa
mRNA expression
(Fold change)
Hsp90
90kDa
siCtl
siCtl
siCtl
DMSO
DMSO
DMSO
siE2F1
siE2F1
siE2F1
HLM006474
HLM006474
HLM006474
E2F1
TP53
C-Myc
MDM2
APAF-1
Bax
Bad
Day 2
Day 3
Day 4
Day 2
Day 3
Day 4
