# Supplementary material for: E2F1 inhibition mediates cell death of metastatic melanoma
Source: Cell Death Dis. 2018 May 9;9(5):527. doi: 10.1038/s41419-018-0566-1 (PMC5943238; doi:10.1038/s41419-018-0566-1)
Supplement: Supplementary file 3 — Supp figure 3 [file 41419_2018_566_MOESM3_ESM.pptx]

## Slide 1
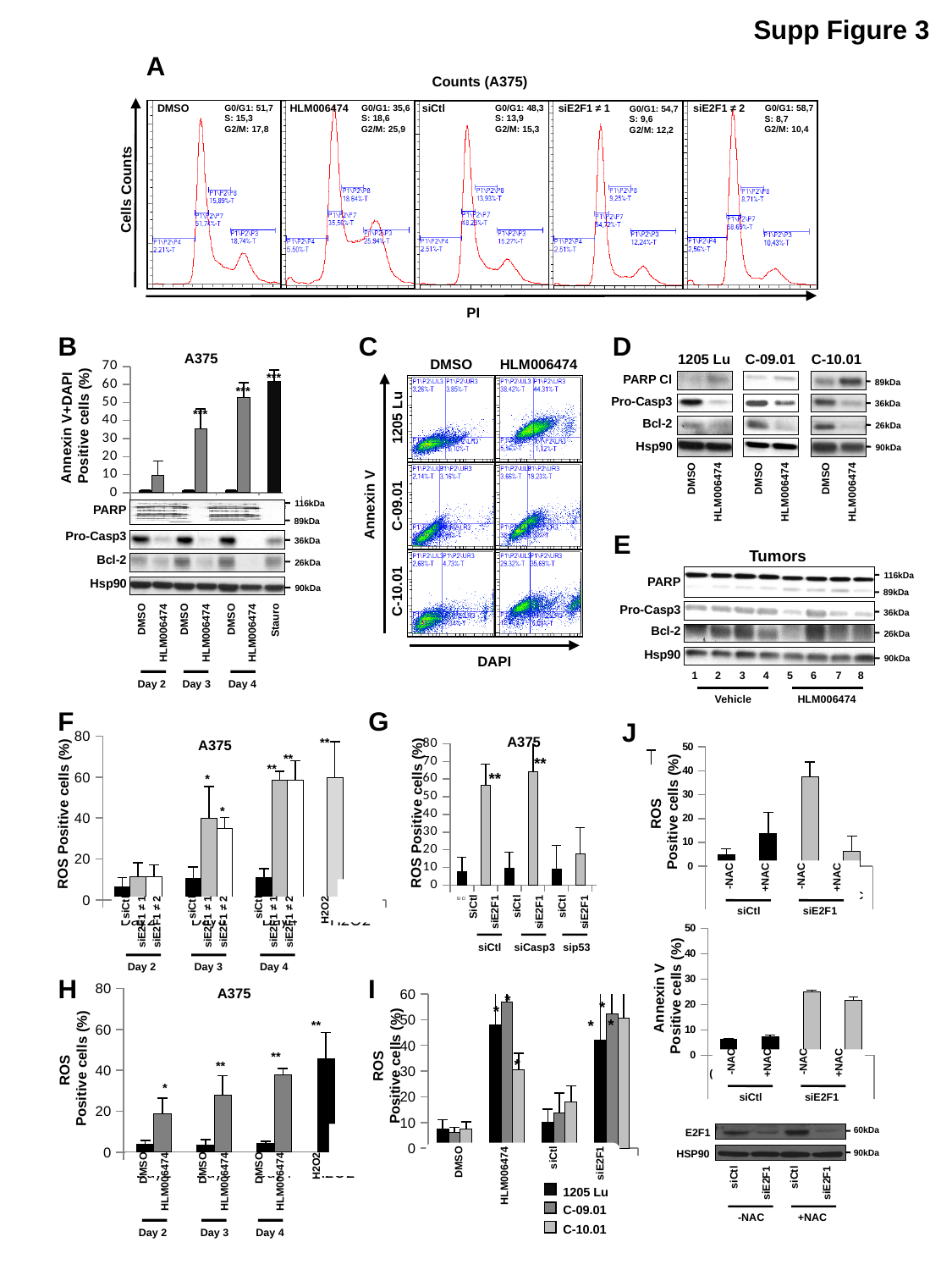

Supp Figure 3
A
Counts (A375)
DMSO
HLM006474
siCtl
siE2F1 ≠ 1
siE2F1 ≠ 2
G0/G1: 35,6
G0/G1: 51,7
G0/G1: 48,3
G0/G1: 58,7
G0/G1: 54,7
S: 18,6
S: 15,3
S: 13,9
S: 8,7
S: 9,6
G2/M: 25,9
G2/M: 17,8
G2/M: 15,3
G2/M: 10,4
G2/M: 12,2
Cells Counts
PI
B
C
D
A375
1205 Lu
C-09.01
C-10.01
[unsupported chart]
DMSO
HLM006474
***
PARP Cl
89kDa
***
Pro-Casp3
36kDa
***
Annexin V+DAPI
Positive cells (%)
1205 Lu
Bcl-2
26kDa
Hsp90
90kDa
DMSO
DMSO
DMSO
HLM006474
HLM006474
HLM006474
Annexin V
C-09.01
116kDa
PARP
89kDa
E
Pro-Casp3
36kDa
Tumors
Bcl-2
26kDa
116kDa
PARP
Hsp90
90kDa
C-10.01
89kDa
Pro-Casp3
36kDa
DMSO
DMSO
DMSO
Stauro
Bcl-2
HLM006474
HLM006474
HLM006474
26kDa
Hsp90
DAPI
90kDa
1
2
3
4
5
6
7
8
Day 2
Day 3
Day 4
HLM006474
Vehicle
F
G
J
[unsupported chart]
A375
**
A375
### Chart
| Category | |
|---|---|
| Si Ctl | 7.732 |
| Si E2F1 | 56.448 |
| Si Ctl + Si Casp3 | 9.747999999999998 |
| Si E2F1 + Si Casp3 | 64.14999999999999 |
| Si Ctl + Si p53 | 9.177999999999999 |
| Si E2F1 + Si p53 | 17.868 |
| Si Ctl + Si p27 | 9.942 |
| Si E2F1 + Si p27 | 19.008 |
| H2O2 | 67.45750000000002 |
### Chart
| Category | % ROS positive Cells |
|---|---|
| (-) NAC | 4.75 |
| (+) NAC | 13.61 |
| (-) NAC | 37.49 |
| (+) NAC | 6.383333333333342 |**
**
**
**
*
ROS
Positive cells (%)
*
ROS Positive cells (%)
ROS Positive cells (%)
-NAC
-NAC
+NAC
+NAC
SiCtl
siCtl
siCtl
siCtl
siCtl
siCtl
H2O2
siE2F1
siCtl
siE2F1
siE2F1
siE2F1
siE2F1 ≠ 1
siE2F1 ≠ 2
siE2F1 ≠ 1
siE2F1 ≠ 2
siE2F1 ≠ 1
siE2F1 ≠ 2
### Chart
| Category | % Annexin V positive Cells |
|---|---|
| (-) NAC | 6.286666666666672 |
| (+) NAC | 7.42666666666667 |
| (-) NAC | 24.963333333333228 |
| (+) NAC | 21.79333333333325 |siCasp3
sip53
siCtl
Day 2
Day 3
Day 4
H
I
Annexin V
Positive cells (%)
[unsupported chart]
A375
### Chart
| Category | | | |
|---|---|---|---|*
*
*
*
*
**
ROS
Positive cells (%)
**
ROS
Positive cells (%)
*
-NAC
-NAC
+NAC
+NAC
**
*
siE2F1
siCtl
60kDa
E2F1
90kDa
HSP90
siCtl
DMSO
siE2F1
H2O2
DMSO
DMSO
DMSO
HLM006474
siCtl
siCtl
HLM006474
HLM006474
HLM006474
siE2F1
siE2F1
1205 Lu
C-09.01
+NAC
-NAC
C-10.01
Day 2
Day 3
Day 4
