# Supplementary material for: E2F1 inhibition mediates cell death of metastatic melanoma
Source: Cell Death Dis. 2018 May 9;9(5):527. doi: 10.1038/s41419-018-0566-1 (PMC5943238; doi:10.1038/s41419-018-0566-1)
Supplement: Supplementary file 4 — Supp figure 4 [file 41419_2018_566_MOESM4_ESM.pptx]

## Slide 1
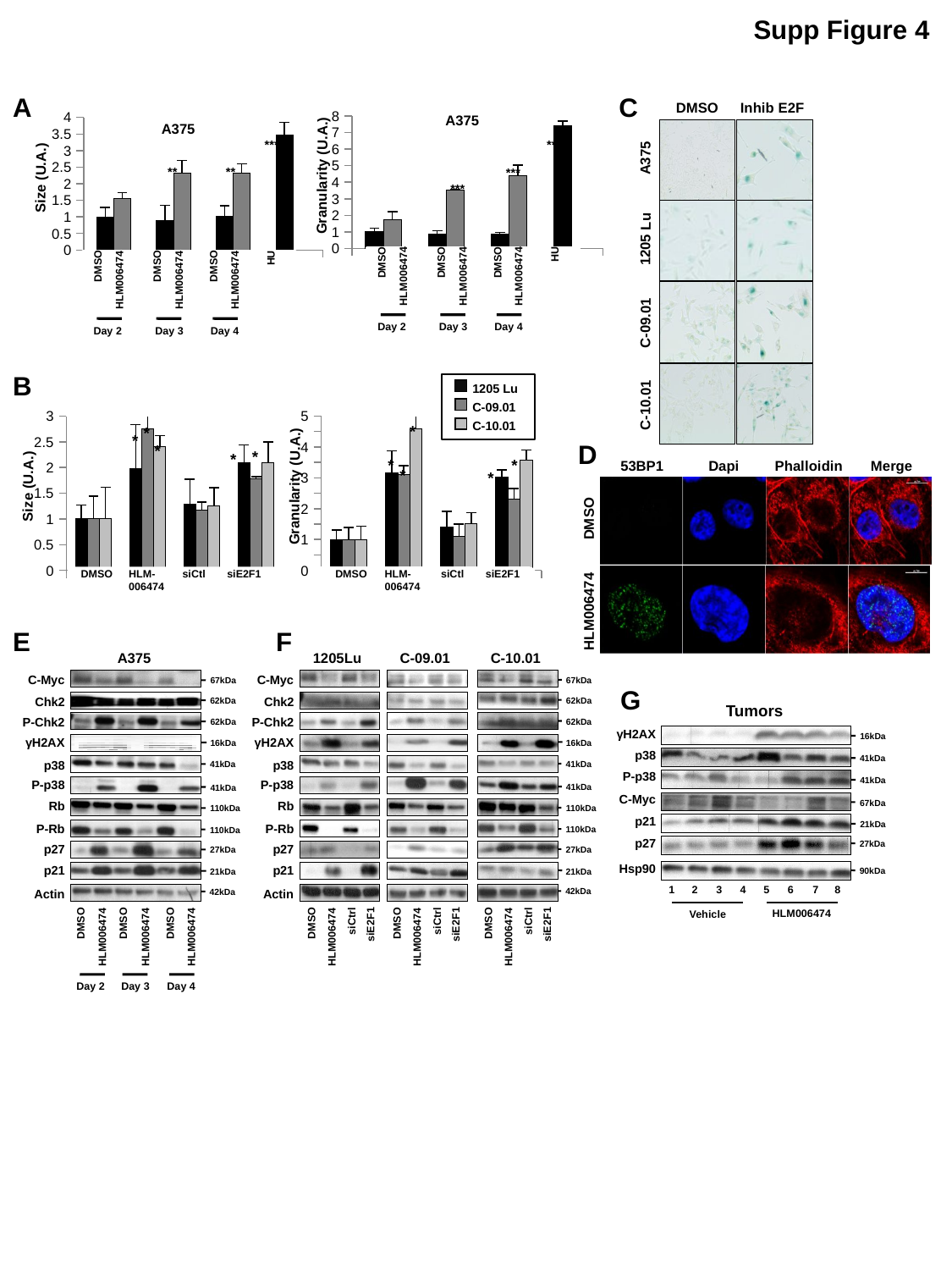

Supp Figure 4
A
C
DMSO
Inhib E2F
### Chart
| Category | DMSO | Inhib E2F |
|---|---|---|A375
### Chart
| Category | DMSO | Inhib E2F |
|---|---|---|A375
***
***
A375
**
**
***
Granularity (U.A.)
Size (U.A.)
***
1205 Lu
HU
HU
DMSO
DMSO
DMSO
DMSO
DMSO
DMSO
HLM006474
HLM006474
HLM006474
HLM006474
HLM006474
HLM006474
C-09.01
Day 2
Day 3
Day 4
Day 2
Day 3
Day 4
B
1205 Lu
C-10.01
C-09.01
### Chart
| Category | | | |
|---|---|---|---|
### Chart
| Category | | | |
|---|---|---|---|C-10.01
*
*
*
D
*
*
*
*
*
53BP1
Dapi
Phalloidin
Merge
*
*
*
Size (U.A.)
Granularity (U.A.)
*
DMSO
DMSO
HLM-
006474
siCtl
siE2F1
DMSO
HLM-
006474
siCtl
siE2F1
HLM006474
E
F
A375
1205Lu
C-09.01
C-10.01
C-Myc
C-Myc
67kDa
67kDa
G
Chk2
Chk2
62kDa
62kDa
Tumors
P-Chk2
P-Chk2
62kDa
62kDa
γH2AX
16kDa
γH2AX
γH2AX
16kDa
16kDa
p38
41kDa
p38
p38
41kDa
41kDa
P-p38
41kDa
P-p38
P-p38
41kDa
41kDa
C-Myc
67kDa
Rb
Rb
110kDa
110kDa
p21
21kDa
P-Rb
P-Rb
110kDa
110kDa
p27
27kDa
p27
p27
27kDa
27kDa
Hsp90
p21
p21
90kDa
21kDa
21kDa
1
2
3
4
5
6
7
8
42kDa
42kDa
Actin
Actin
HLM006474
Vehicle
siCtrl
siCtrl
siCtrl
DMSO
DMSO
DMSO
DMSO
DMSO
DMSO
siE2F1
siE2F1
siE2F1
HLM006474
HLM006474
HLM006474
HLM006474
HLM006474
HLM006474
Day 2
Day 3
Day 4
