# Supplementary material for: E2F1 inhibition mediates cell death of metastatic melanoma
Source: Cell Death Dis. 2018 May 9;9(5):527. doi: 10.1038/s41419-018-0566-1 (PMC5943238; doi:10.1038/s41419-018-0566-1)
Supplement: Supplementary file 5 — Supp figure 5 [file 41419_2018_566_MOESM5_ESM.pptx]

## Slide 1
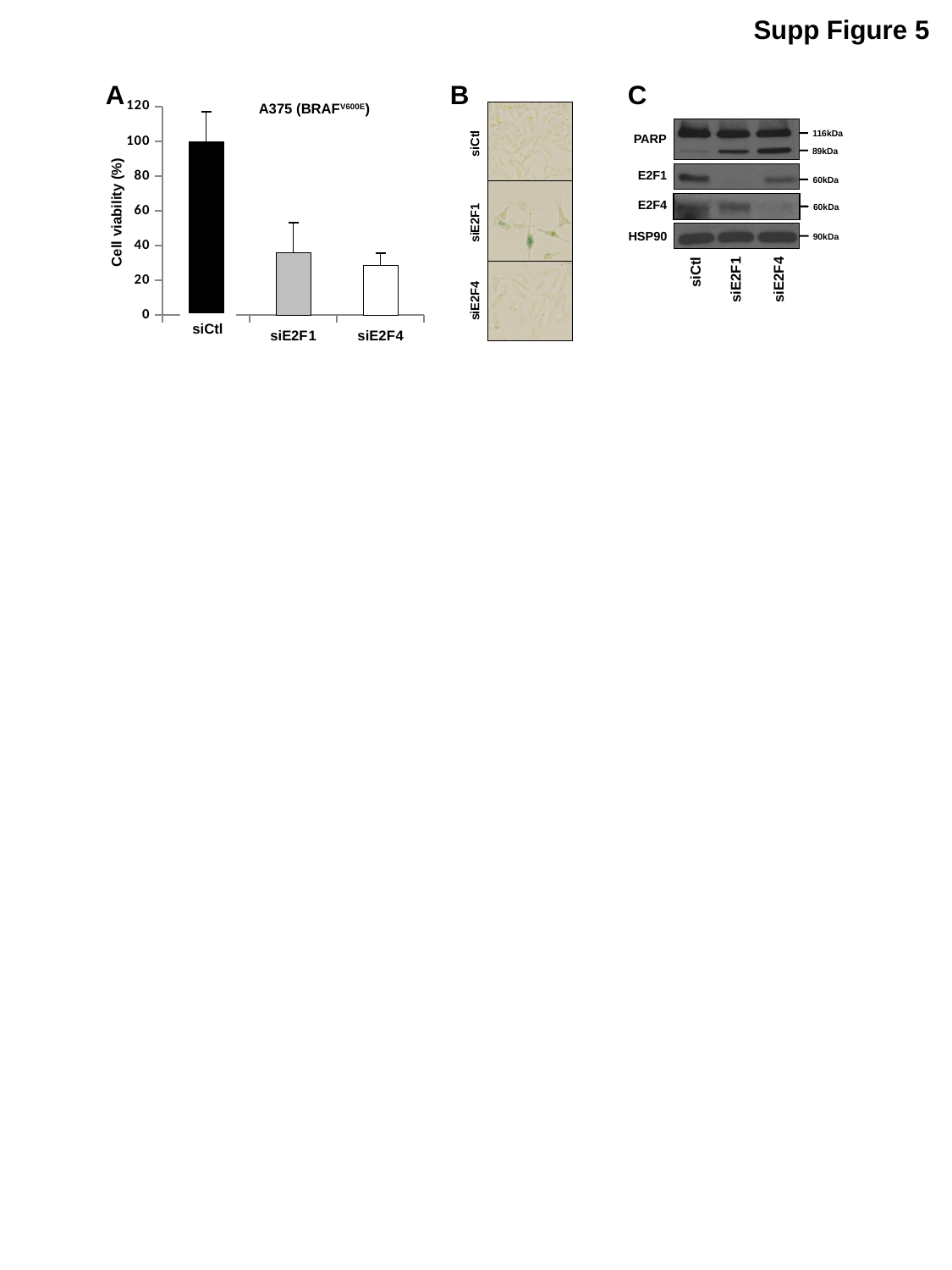

Supp Figure 5
A
B
C
A375 (BRAFV600E)
### Chart
| Category | |
|---|---|
| siCTR | 100.0 |
| siE2F1 | 36.01532567049808 |
| siE2F4 | 28.35249042145594 |Cell viability (%)
116kDa
PARP
siCtl
89kDa
E2F1
60kDa
E2F4
60kDa
siE2F1
HSP90
90kDa
siCtl
siE2F1
siE2F4
siE2F4
siCtl
