# Supplementary material for: E2F1 inhibition mediates cell death of metastatic melanoma
Source: Cell Death Dis. 2018 May 9;9(5):527. doi: 10.1038/s41419-018-0566-1 (PMC5943238; doi:10.1038/s41419-018-0566-1)
Supplement: Supplementary file 6 — Supp figure 6 [file 41419_2018_566_MOESM6_ESM.pptx]

## Slide 1
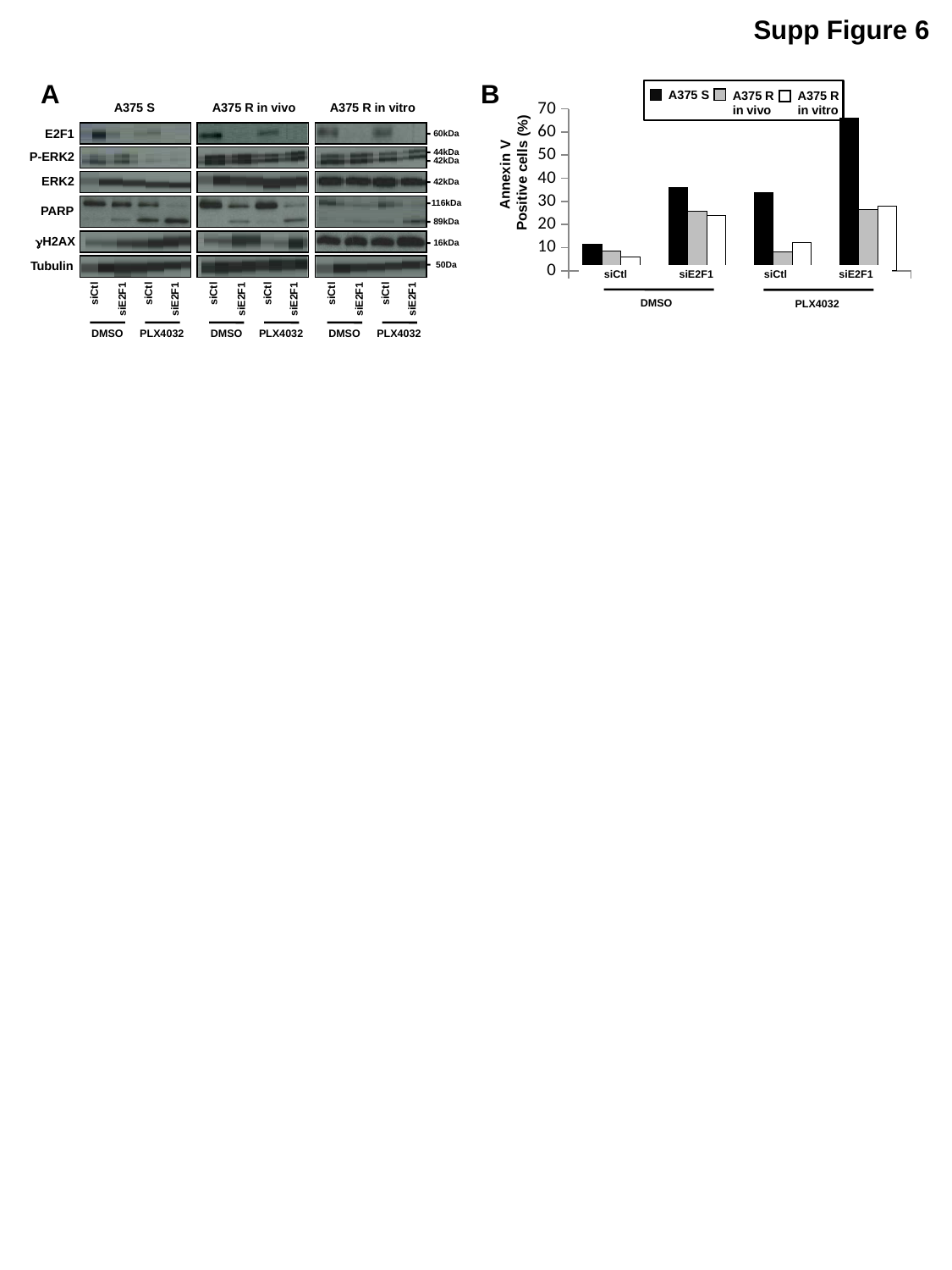

Supp Figure 6
A
B
A375 S
A375 R
in vivo
A375 R
in vitro
A375 S
A375 R in vivo
A375 R in vitro
### Chart
| Category | | | |
|---|---|---|---|E2F1
60kDa
44kDa
P-ERK2
Annexin V
Positive cells (%)
42kDa
ERK2
42kDa
116kDa
PARP
89kDa
γH2AX
16kDa
Tubulin
50Da
siCtl
siE2F1
siCtl
siE2F1
siCtl
siCtl
siCtl
siCtl
siCtl
siCtl
siE2F1
siE2F1
siE2F1
siE2F1
siE2F1
siE2F1
DMSO
PLX4032
PLX4032
PLX4032
PLX4032
DMSO
DMSO
DMSO
