# Supplementary material for: E2F1 inhibition mediates cell death of metastatic melanoma
Source: Cell Death Dis. 2018 May 9;9(5):527. doi: 10.1038/s41419-018-0566-1 (PMC5943238; doi:10.1038/s41419-018-0566-1)
Supplement: Supplementary file 7 — Supplemental materials [file 41419_2018_566_MOESM7_ESM.docx]

**Supplemental Materials and methods**

Resistant melanoma cell lines A375 and WM9

Briefly, to generate cell lines with in vitro acquired resistance, BRAFV600E mutant cell lines (A375, WM9) sensitive to vemurafenib, were treated with increased concentrations of vemurafenib for 2-3 months until a subline grew progressively. Vemurafenib-resistant cells were cloned in 3μM vemurafenib, a concentration at which parental cells were not viable. Vemurafenib-resistant cells obtained *in vitro* were designated A375 R *in vitro*. Trypan blue exclusion assay was regularly performed to check resistance status. To obtain the A375 R *in vivo* vemurafenib-resistant cell line, we used human melanoma xenograft models in which drug resistance was selected by continuous vemurafenib administration in immunocompromised mice. Briefly, A375 orthotopic tumors were grown to 300 mm^3^ before treatment with PLX4032 (75mg/kg/day by oral gavage). Tumor growth was inhibited for 45 days of treatment, at which time one tumor rapidly progressed. The occurrence of resistance is in line with clinical data in humans. Once this tumor reached a volume of 1500 mm^3^, the mouse was euthanized, tumor tissue removed and primary cell culture was established. The resistant melanoma cells were maintained in culture in presence of 3 μM of PLX4032.

**Supplemental Results**

Since the production of ROS represents one of the first events in p53-regulated apoptosis, we analyzed the level of ROS in melanoma cells by flow cytometry using a CellROX® Deep Red probe. In our model, the inhibition of E2F1 increased the production of ROS (Supp Fig.3F and Supp. Fig.3H and 3I), suggesting that ROS production could be an important process involved in E2F1 inhibition-mediated apoptosis.

To confirm the implication of p53 in the production of ROS mediated by E2F1 inhibition, we analyzed levels of ROS and we observed that the co-inhibition of p53 and E2F1 blocked the production of ROS induced by inhibition of E2F1 alone (Supp Fig.3G). Collectively, these results indicate that the loss of E2F1 triggers p53-dependent apoptosis associated with decreased viability of melanoma cells.

**Supplemental figure legends**

**Supplemental Figure 1: Inhibition of E2F1 by an inhibitor aspecific a HLM006474 decreases cell viability in melanoma cells and inhibits tumor growth in a xenograft model.**

A. A375 melanoma cells were treated with 20 µM of E2F inhibitor (HLM006474). At the indicated times, viable cells were counted using the trypan blue dye exclusion method. In parallel, cells were lysed and analyzed by western blotting using the indicated antibodies. HSP90 was used as a loading control.

B. Indicated melanoma cell lines or human melanoma cells freshly isolated from tumors were treated with 20 µM of HLM006474. After 4 days, viable cells were counted using the trypan blue dye exclusion method.

For (A–B), the results are expressed as percentage of control and data are means ± SD of three independent experiments performed in triplicate. *p < 0.05; **p < 0.01; ***p < 0.001.

C. RNAs were extracted from A375 cells transfected with 50 nM siCTRL or siE2F1 or treated with DMSO or HLM006474 for 2 days. Different mRNA expression was measured by SYBR green-based real-time Q-PCR and averaged from three independent experiments. The relative expression level of mRNA was normalized to SB34.

D. A375 melanoma cells were transfected with siCtl or 2 different siE2F1 for 2 days. Cells were lysed and analyzed by western blotting using the indicated antibodies. HSP90 was used as a loading control.

E. Survival of melanoma patients with high and low (above or below the median, respectively) mRNA levels of E2F4. Gene expression data of 44 metastatic melanoma tissues ^13^ were used to define high and low expressor groups (boxplots, Mann-Whitney test) and to generate Kaplan-Meier curves (log-rank test).

F. Primary human melanocytes and keratinocytes were treated for 4 days with 20 µM of HLM006474. Cell viability was estimated by trypan blue staining. The results are expressed as percentage of control and data are means ± SD of three independent experiments performed in triplicate.

G. Female immune-deficient BALB/c nu/nu (nude) mice were inoculated subcutaneously with 1.5x10^6^ A375 melanoma cells. After 6 days, mice (n = 6 in each group) were treated with HLM006474 (2 mg/mouse/day for 8 days) or vehicle. The tumor growth curves were determined by measuring the tumor volume. The bars indicate the mean ± SD. *p < 0.05; **p < 0.01; ***p < 0.001.

H. At the end of the experiment show in (D), mice were euthanized and tumors were weighed. The bars indicate the mean ± SD. ***p < 0.001. In parallel, tumors were lysed and analyzed by western blot using the indicated antibodies. HSP90 was used as a loading control.

**Supplemental Figure 2: HLM006474 increases p53 level *in vitro* and *in vivo***

A. Melanoma cell lines A375 and 1205 Lu or human melanoma cells freshly isolated from tumors were treated with 20 µM of HLM006474 for 4 days. p53 levels were determined by western blot analysis. Hsp90 was used as loading control.

B. Mice tumors were lysed and analyzed by western blot to determine p53 levels. Hsp90 was used as loading control.

C. Immunofluorescence pictures of A375 melanoma cells treated with 20 µM of HLM006474. p53 was labeled with specific antibody (magenta), DNA was visualized with DAPI (blue) and phalloidin was visualized in red.

D. A375 melanoma cells were transfected with siCtl or siE2F1 or treated with HLM006474 for indicated times. At the end of experiment, cells were lysed and analyzed by western blotting to determine MDM2 levels. Hsp90 was used as loading control.

E. RNAs were extracted from A375 cells transfected with siCtl or siE2F1 or treated with DMSO or HLM006474 for 3 days. Different mRNA expression was measured by SYBR green-based real-time Q-PCR and averaged from three independent experiments. The relative expression level of mRNA was normalized to GAPDH and normalized to siCtl.

**Supplemental Figure 3: HLM006474 induces apoptosis *in vitro* and *in vivo***

A. A375 melanoma cells were transfected with siCtl or siE2F1 or treated with HLM006474 for 2 days. At the end of experiment, cells were stained with PI to detect cell cycle phase by flow cytometry.

B. A375 melanoma cells were treated with 20 µM of HLM006474 for indicated times. At the end of experiment, cells were co-stained with Annexin V and DAPI to detect cell dead by flow cytometry. Staurosporine was used as positive control. In parallel, cells were lysed and analyzed by western blotting using the indicated antibodies. HSP90 was used as a loading control.

C. D. 1205Lu melanoma cells and human melanoma cells freshly isolated from tumors were treated with 20 µM of HLM006474. C. Cells were co-stained with Annexin V and DAPI to detect cell dead by flow cytometry. D. In parallel, cells were lysed and analyzed by western blotting using the indicated antibodies. HSP90 was used as a loading control.

E. Mice tumors were lysed and analyzed by western blot to determine the different markers of apoptosis. Hsp90 was used as loading controls.

F. G. H. I. The percentage of ROS formed was analyzed by flow cytometry using Cell Rox Deep Red. F. A375 melanoma cells transfected with siCtl or siE2F1 for indicated times. G. A375 melanoma cells were transfected with siCasp3 or sip53 and co-transfected with siCtl or siE2F1 for 4 days. H, A375 melanoma cells treated with 20 µM of HLM006474 for indicated times. I, Melanoma cell lines 1205 Lu or human melanoma cells freshly isolated from tumors were treated with 20 µM of HLM006474 for 4 days.

J. A375 melanoma cells were pre-treated with NAC (N-acetyl-L-cysteine) and cells were transfected with siCtl or siE2F1 for 3 DAYS. The percentage of ROS formed was analyzed by flow cytometry using Cell Rox Deep Red. Cells were stained with Annexin V to detect apoptosis by flow cytometry. In parallel, cells were lysed and analyzed by western blotting using the indicated antibodies. HSP90 was used as a loading control.

For (B, F-J), the results are expressed as percentages of the control. Data are means ± SD of three independent experiments. *p < 0.05; **p < 0.01; ***p < 0.001.

**Supplemental Figure 4: Inhibition of E2F1 by siRNA or HLM006474 induces senescence and DNA damage.**

A. A375 melanoma cells were treated with 20 µM of HLM006474 for indicated times. Cells were then analyzed by flow cytometry for the relative size and cell granularity. Hydroxyurea (HU) was used as positive control.

B.1205 Lu melanoma cell lines or human melanoma cells freshly isolated from tumors were treated with 20 μM of HLM006474 or transfected with siE2F1 (50nM) for 4 days. Cells were then analyzed by flow cytometry for the relative size and cell granularity.

For (A-B) the results are expressed as percentage of control. Data are means ± SD of three independent experiments performed in triplicate. *p < 0.05; **p < 0.01; ***p < 0.001.

C. Indicated melanoma cell lines or human melanoma cells freshly isolated from tumors were treated with 20 µM of HLM006474. After 4 days, cells were stained for SA-β-Gal activity. A representative picture was shown.

D. Immunofluorescence pictures of A375 melanoma cells treated with 20 µM of HLM006474 for 4 days. 53BP1 was labeled with specific antibody (green), DNA was visualized with DAPI (blue) and phalloidin was visualized with red.

E. A375 melanoma cells treated with 20 µM of HLM006474 for indicated times. Melanoma cells lysates were analyzed by western blotting using the indicated antibodies. Actine or HSP90 were used as a loading control.

F. 1205 Lu melanoma cell lines or human melanoma cells freshly isolated from tumors were treated with 20 µM of HLM006474 or transfected with siE2F1 for 4 days. Melanoma cells lysates were analyzed by western blotting using the indicated antibody. Actine was used as a loading control.

G. Mice tumors were lysed and analyzed by western blot using the indicated antibodies. HSP90 was used as a loading control.

**Supplemental Figure 5: Inhibition of E2F4 by siRNA induces apoptosis but not senescence.**

A. B. C. A375 melanoma cells were transfected with siCtl or siE2F1 or siE2F4 (50nM) for 4 days. A. viable cells were counted using the trypan blue dye exclusion method. The results are expressed as percentage of control. B. Cells were stained for SA-β-Gal activity. C. Melanoma cells lysates were analyzed by western blotting using the indicated antibodies. HSP90 was used as a loading control.

**Supplemental Figure 6: Co-Inhibition of E2F1 by siRNA and BRaf by PLX4032 induces more apoptosis of melanoma cells sensitive but not resensitive melanoma cells resistant at PLX4032.**

A-B. A375 melanoma cells were transfected with siCtl or siE2F1 and 36h later, cells were treated with BRAF inhibitors, PLX4032 (5 µM) for 48h. A. cells were lysed and analyzed by western blotting using the indicated antibodies. HSP90 was used as a loading control. B. Cells were stained with AnnexinV to detect apoptosis cells by flow cytometry.
